# Proteomics Analysis of Human Obesity Reveals the Epigenetic Factor HDAC4 as a Potential Target for Obesity

**DOI:** 10.1371/journal.pone.0075342

**Published:** 2013-09-24

**Authors:** Mohamed Abu-Farha, Ali Tiss, Jehad Abubaker, Abdelkrim Khadir, Fahad Al-Ghimlas, Irina Al-Khairi, Engin Baturcam, Preethi Cherian, Naser Elkum, Maha Hammad, Jeena John, Sina Kavalakatt, Samia Warsame, Kazem Behbehani, Said Dermime, Mohammed Dehbi

**Affiliations:** 1 Department of Biomedical Research, Dasman Diabetes Institute, Kuwait, Kuwait; 2 Fitness and Rehabilitation Centre, Dasman Diabetes Institute, Kuwait, Kuwait; 3 Department of Biostatistics & Epidemiology, Dasman Diabetes Institute, Kuwait, Kuwait; 4 Biomedical Research Facility, King Fahad Specialist Hospital Dammam, Dammam, Kingdom of Saudi Arabia; 5 Genomic Medicine and Systems Biology Research Center, Qatar Biomedical Research Institute, Education City, Doha, Qatar; Charité-University Medicine Berlin, Germany

## Abstract

Sedentary lifestyle and excessive energy intake are prominent contributors to obesity; a major risk factors for the development of insulin resistance, type 2 diabetes and cardiovascular diseases. Elucidating the molecular mechanisms underlying these chronic conditions is of relevant importance as it might lead to the identification of novel anti-obesity targets. The purpose of the current study is to investigate differentially expressed proteins between lean and obese subjects through a shot-gun quantitative proteomics approach using peripheral blood mononuclear cells (PBMCs) extracts as well as potential modulation of those proteins by physical exercise. Using this approach, a total of 47 proteins showed at least 1.5 fold change between lean and obese subjects. In obese, the proteomic profiling before and after 3 months of physical exercise showed differential expression of 38 proteins. Thrombospondin 1 (TSP1) was among the proteins that were upregulated in obese subjects and then decreased by physical exercise. Conversely, the histone deacetylase 4 (HDAC4) was downregulated in obese subjects and then induced by physical exercise. The proteomic data was further validated by qRT-PCR, Western blot and immunohistochemistry in both PBMCs and adipose tissue. We also showed that HDAC4 levels correlated positively with maximum oxygen consumption (V_O2 Max_) but negatively with body mass index, percent body fat, and the inflammatory chemokine RANTES. In functional assays, our data indicated that ectopic expression of HDAC4 significantly impaired TNF-α-dependent activation of NF-κB, establishing thus a link between HDAC4 and regulation of the immune system. Together, the expression pattern of HDAC4 in obese subjects before and after physical exercise, its correlation with various physical, clinical and metabolic parameters along with its inhibitory effect on NF-κB are suggestive of a protective role of HDAC4 against obesity. HDAC4 could therefore represent a potential therapeutic target for the control and management of obesity and presumably insulin resistance.

## Introduction

The increase in sedentary lifestyles and excessive food intake are considered as key contributing factors to a group of obesity-associated disorders such as insulin resistance, diabetes, dyslipidemia and cardiovascular complications that substantially contribute to a significant reduction of either life quality or expectancy, if not both [1]. Paradoxically coexisting in developing countries with under-nutrition, obesity has become a key risk factor contributing to the overall burden of chronic disease occurring over a wide geographic area and affecting virtually all ages, genders and socioeconomic groups [2]. The recent annual report from the International Association for the Study of Obesity documented approximately 1.5 billion of adults that are overweight, of whom around 525 million are clinically obese (www.iaso.org). In Kuwait, the prevalence of overweight and obesity is highly alarming among adult population with rates of 80.4% and 47.2%, respectively [3]. Exercise is an important component of healthy lifestyle that has long-time been prescribed as a part of the prophylactic treatment and management of obesity, insulin resistance and their associated complications [4].

Obesity is characterized by a chronic, low-grade inflammatory response referred to as metaflammation and dysregulation of the stress response system in key metabolic organs such as liver, muscle, adipose tissue and pancreas [5]. The stress response; known also as metabolic stress, is highly complex and includes an impairment of the heat shock response [6,7], excessive activation of the oxidative stress versus a down-regulation of the cellular antioxidant defence system [8,9], dysfunction of the mitochondria or defect in its biogenesis [10], hypoxia [11], and the emerging role of the endoplasmic reticulum (ER)-mediated stress [12]. Recent investigations indicated that the inflammatory and stress responses pathways are highly integrated and they work most-likely in vicious cycles, which largely explain the myriad of disorders associated with obesity [13,14]. Metabolic stresses are known to activate several stress kinases but the best characterized are the c-Jun N-terminal kinase (JNK) and the inhibitor of κB kinase-β (IKKβ), both of them can phosphorylate the insulin receptor substrate-1 (IRS-1) and rendering this crucial intermediate a poor substrate for the activated insulin receptor [15,16].

Several genomic and proteomic studies have been carried over the last decade on human and mouse adipose tissue and the outcomes of such efforts were pivotal in gaining insight into the molecular mechanisms underlying obesity and its associated complications [17,18,19,20,21]. A recent proteomics study was carried out on two-major intra-abdominal human fat tissues, namely the subcutaneous and the omental fat to identify and validate obesity-related reference proteins for data standardization [22]. Accordingly, only ENOA, PARK7 and β-actin proteins were reported as proper reference standards for omental fat in obesity studies, while FAA was shown to be the best control for both omental and subcutaneous adipose tissues regardless of the obesity status [22]. The limited number of identified proteins was, in large part due to the inherent limitation of the technology (2D gel combined to Maldi-Tof) and the technical challenges to extract soluble proteins from fat adipose tissue in sufficient amounts.

In the present study, we applied a shot-gun proteomic profiling approaches on PBMCs isolated from lean and obese human male subjects to identify and quantify proteins expressed in the two groups and established their possible correlation with clinical outcomes. This study will provide a snapshot of the proteomic profile of lean and obese subject and the data generated can be used to formulate novel hypotheses on the molecular mechanisms orchestrating obesity and its management with physical exercise. PMBCs were chosen as surrogate tissue in this investigation based on their important inflammatory and stress response roles in a variety of diseases including obesity. Based on the beneficial effect of physical exercise on improving the clinical outcomes associated with obesity; we also investigated its impact on differentially expressed proteins in obese subjects.

Using this approach, 47 proteins were found to be differentially expressed between lean and obese subjects. Proteins that were upregulated in obese group included thrombospondin 1 (TSP1), whereas, the histone deacetylase 4 (HDAC4) protein levels were reduced in obese subjects. In obese subjects, the proteomic profiling before and after 3 months of physical exercise showed differential expression of 38 proteins. Interestingly, the expression levels of both TSP1 and HDAC4 were corrected by physical exercise and became comparable to the levels initially observed in control group. In functional assays, our data indicated that overexpression of HDAC4 significantly impaired TNF-α-dependent activation of NF-κB, establishing thus a link between HDAC4 and regulation of the immune system. Together, our *in vivo* and *in vitro* data on HDAC4 suggest that it has a protective role against obesity and could therefore represent a potential therapeutic target for management of obesity and presumably in insulin resistance and type 2 diabetes.

## Materials and Methods

### Study population and ethical statement

The current investigation is part of a large cohort study aimed at investigating the effect of physical exercise on the inflammatory, metabolic and stress responses in obese and diabetic subjects. In the present study, we selected 48 adult non-diabetic male participants consisting of 11 lean (20 ≤ BMI < 25 kg/m^2^) and 37 obese (30 ≤ BMI < 40 kg/m^2^) to identify differentially expressed proteins between the two groups. Written informed consent was obtained from all subjects before their participation in the study which was approved by the Review Board of Dasman Diabetes Institute and carried out in line with the guideline ethical declaration of Helsinki. All volunteers were subjected to a pre-screening assessment to determine their eligibility to participate in the study. Glucose and lipid profiles were analyzed for all volunteers. Candidates that followed any physical exercise within the last 6 months prior to this study, morbid obese (i.e. BMI >40 kg/m^2^) and those with prior major illness were excluded from the study. In addition, eligible candidates should not be taking any medication and/or supplement known to influence the body composition or bone mass. The physical as well as the clinical and biochemical parameters of the participating subjects are shown in Table 1 and Table 2, respectively.

**Table 1:** Physical characteristics of the study population at baseline.

	**Lean (n = 11**)	**Obese (n = 37**)	***P-value***
Age (year)	42.73 ± 9.71	46.38 ± 12.50	*0.37*
BMI (kg/m^2^)	23.67 ± 1.47	34.13 ± 2.82	*<0.0001*
PBF (%)	24.21 ± 2.27	34.65 ± 3.26	*<0.0001*
Waist (cm)	82.35 ± 16.67	113.34 ± 8.53	*<0.0001*
Hip (cm)	93.55 ± 6.07	113.92 ± 7.62	*<0.0001*

Data are presented as mean ± SD. Mann-Whitney t-test was used to compare differences between lean and obese subjects. BMI (body mass index), PBF (percent body fat).

**Table 2:** Clinical and biochemical parameters of the study population at baseline.

	**Lean (n=11**)	**Obese (n=37**)	***P-value***
Resting HR (beat/min)	86.67 ± 19.54	73.78 ± 7.60	*0.1*
SBP (mmHg)	113.33 ± 12.11	131.67 ± 12.25	*0.03*
DBP (mmHg)	76.67 ± 5.16	86.00 ± 9.66	*0.05*
V_O2 Max_ (ml/kg/min)	23.34 ± 3.21	18.8 ± 5.35	*0.11*
Cholesterol (mmol/l)	5.00 ± 0.59	5.12 ± 1.04	*0. 76*
HDL (mmol/l)	1.34 ± 0.78	1.04 ± 0.22	*0.16*
LDL (mmol/l)	3.07 ± 1.00	3.38 ± 0.92	*0.59*
TG (mmol/l)	1.14 ± 0.52	1.52 ± 80	*0.13*
Glucose (mmol/l)	5.20 ± 0.60	5.59 ± 0.95	*0.29*
HbA1C (%)	5.56 ± 0.55	5.95 ± 0.64	*0.07*
C-peptide (ng/ml)	2.79 ± 0.74	3.54 ± 1.35	*0.18*
GLP-1 (ng/ml)	2.50 ± 0. 91	2.91 ± 1.60	*0.96*
Insulin (ng/ml)	2.55 ± 1.23	3.97 ± 2.21	*0.08*
Leptin (ng/ml)	3.19 ± 1.37	6.60 ± 2.69	*0.001*
PAI-1 (ng/ml)	2.45 ± 0.72	3.98 ± 1.14	*0.006*
TNF-α (pg/ml)	18.15 ± 1.86	31.91 ± 14.95	*0.13*
IL-1β (pg/ml)	1.10 ± 0.46	1.28 ± 0.48	*0.34*
IL-6 (pg/ml)	3.89 ± 1.20	5.41 ± 2.48	*0.08*
IL-10 (pg/ml)	1.31 ± 0.99	2.43 ± 1.84	*0.17*
IP-10 (ng/ml)	0.39 ± 0.19	0.55 ± 0.22	*0.07*
RANTES (ng/ml)	1.12 ± 0.42	1.61 ± 0.68	*0.04*
ROS (mM)	1.43 ± 0.35	1.44 ± 0.14	*0.83*
TBARS (μM)	1.01 ± 0.45	1.64 ± 0.54	*0.006*

Data are presented as mean ± SD. Mann-Whitney t-test was used to compare differences between lean and obese subjects. HR (heart rate), SBP (systolic blood pressure), DBP (diastolic blood pressure), V_O2 Max_ (maximum oxygen consumption), HDL (high density lipoprotein), LDL (low density lipoprotein) and TG (triglycerides).

### Exercise Program

All eligible subjects were enrolled to a supervised exercise program at the Fitness and Rehabilitation Center (FRC) of Dasman Diabetes Institute. Prior to exercise prescription, each individual underwent a symptom-limited maximal incremental cardiopulmonary exercise test “CPET” (COSMED Quark, Italy) using an electromagnetically braked cycle ergometer. The CPET was primarily used to determine the maximum heart rate (max HR) as well as the response to aerobic exercise as measured by the maximum oxygen consumption (V_O₂ Max_) for each subject. Thereafter, a physical fitness assessment test was performed to determine muscle strength and endurance along with flexibility by performing grip strength (dynamometer), push-ups (upper body strength), sit-ups and forward bending test (both upper and lower body flexibility). The exercise training involves a combination of both moderate intensity of aerobic exercise and resistance training using either treadmill or cycling. Each exercise session includes 10 minutes warming-up and cooling down steps at 50-60% of max HR, along with 40 minutes of the prescribed exercise program at 65-80% of max HR. For the duration of the 3-months period, participants exercised 3 to 5 times per week and they were instructed to reach and maintain the recommended heart rate range. This was achieved by regular monitoring of the heart rate during the aerobic training. Strength training was performed 2 to 3 times a week according to the program plan. Exercise intensity, duration and blood pressures were recorded for each session. All trainings were supervised by qualified fitness professionals and a consultant respirologist from FRC. To assess the effectiveness of the exercise, the same physical stress and fitness tests were performed for all subjects at the end of the exercise program.

### Anthropometric and biochemical measurements

Anthropometric measurements were taken at the baseline and after 3 months of exercise. Whole-body composition was determined by dual-energy radiographic absorptiometry device (Lunar DPX, Lunar radiation, Madison, WI). Glucose and lipid profiles were measured on the Siemens Dimension RXL chemistry analyzer (Diamond Diagnostics, Holliston, MA). HbA1C was determined using the Variant^TM^ device (BioRad, Hercules, CA). Plasma levels of inflammatory and metabolic markers were measured using bead-based multiplexing xMAP® technology (Luminex, Austin, TX) using the Bio-Plex Pro™ human cytokine 27-plex assay and diabetes 10-plex assay kits (BioRad, Hercules, CA). Median fluorescence intensities were collected on a Bioplex-200 system using Bio-plex Manager software version 6 (BioRad, Hercules, CA). Lipid peroxidation was assessed by measuring plasma levels of malonaldehyde, using TBARs assay kit (Cayman Chemical Company, Ann Arbor, MI). Serum levels of ROS were determined using the OxiSelect™ ROS Assay Kit (Cell Biolabs Inc, San Diego, CA). All the above assays were carried out according to the instructions of the manufacturers.

### Blood and tissue sampling

Venous peripheral blood and subcutaneous adipose tissue biopsies were obtained before starting the exercise (baseline) and after the 3 months of exercise period. Peripheral blood mononuclear cells (PBMCs) were prepared from 40 ml of blood using Ficoll-Hypaque density gradient centrifugation method and then, resuspended in freezing media containing 10% DMSO and stored in liquid nitrogen. Plasma and serum were prepared using vacutainer tubes and then aliquoted and stored at -80°C until assayed. Subcutaneous superficial adipose tissue biopsies (~0.5 g) were obtained by a surgeon from the periumbilical area by surgical biopsy after a local anesthesia. Once removed, the biopsy was rinsed in cold PBS, divided into 4 pieces and stored appropriately until used for mRNA extraction and immunohistochemical studies.

### Preparation of protein extract for MS analysis

Frozen PBMCs collected from lean subjects (n=6), obese subjects before (n=6) and after exercise (n=6) were washed with ice-cold PBS and the total cell count and viability were determined by using Countess™ Automated Cell Counter as instructed by the manufacturer (Invitrogen, Carlsbad, CA). Equal amount of cells (2.5x10^6^) were pooled to make three pairs for each group and then, used to prepare whole cell extracts. After centrifugation, the cell pellet was resuspended in 200 µL of lysis buffer (6M urea, 4% CHAPS) supplemented with a mini complete protease inhibitor cocktail (Roche Diagnostics, Laval, Quebec) for 30 min at 4°C. Extracts were centrifuged at 14,000 rpm for 30 minutes at 4°C. Protein concentration was determined by Bradford method using γ-globulin as a standard and proteins were subjected to centrifugal proteomic reactor as described previously [23]. Briefly, 20 µg of proteins were added to 10 µL of strong cationic exchange (BcMag™ SCX Magnetic bead slurry (Bioclone, San Diego, CA) in 1 ml of 5% formic acid and the mixture was vigorously vortexed. After 2 min centrifugation at 10,000 g, the pellet was resuspended in 1 ml of 0.5% formic acid and then, samples were reduced by adding 20 µl of reducing solution (150 mM NH_4_HCO_3_, 20 mM DTT) for 15 min at 56°C with constant rotation at 800 rpm and then alkylated by adding 20 µl of alkylating solution (150 mm NH_4_HCO_3_, 100 mm iodoacetamide) for 15 min at room temperature. The alkylation reaction was stopped by adding 1 ml of 0.5% formic acid. The beads were centrifuged and proteins were digested with trypsin for overnight at 37°C with constant shaking and then, eluted with 1 ml of 5% formic acid at four different pH levels (2.5, 4, 8 and 12). Fractions were lyophilized to complete dryness in a speed-vac and stored at -20°C until subjected to MS analysis.

### NanoLC-MS/MS Analysis

The analysis of peptide digests was done by Liquid chromatography (Easy nanonLC; Proxeon Biosystems, Denmark) coupled to tandem mass spectrometry (MS/MS; LTQ-Orbitrap Velos, Thermo Scientific, Germany). Briefly, peptides samples were first dissolved in 5% formic acid and then loaded on a C18-A1 easy column (Proxeon Biosystems, Denmark). Peptides were desalted with 5% acetonitrile/ 0.1% formic acid before their elution from the C18-A1 column. The peptides were then directed to a C18-A2 analytical easy column (Proxeon Biosystems, Denmark). Peptides were finally eluted at a gradient of 5 to 35% acteonitrile with 0.1% formic acid over 80 min and analyzed with MS.

The full MS spectra scan was performed at a resolution of 60,000. MS/MS spectra were acquired in a data-dependant acquisition mode that automatically selected and fragmented the fifteen most intense peaks from each MS spectrum generated. Raw data files were analyzed using the Proteome Discoverer 1.3 software (Thermo Scientific; Germany) using Sequest and Mascot search engine against the *Homo sapiens* International Protein Index (IPI) protein sequence database version 3.68 (European Bioinformatics Institute, United Kingdom). Search parameters were set as follow: trypsin was selected as digestive enzyme with 2 miss-cleavages allowed, carbamidomethyl of cysteine as a fixed modification and methionine oxidation as a variable modification. Peptide and MS/MS mass tolerances were set at 7 ppm and 0.8 Da, respectively. Peptide confidence was set to high ensuring a 1% false discovery rate (FDR). Sieve software version 1.3 (Thermo Scientific, Germany) was used for quantification of proteins identified. Molecular functions and protein networks were analyzed using the Ingenuity pathways analysis (IPA) software (Ingenuity Systems; Qiagen, Inc., Valencia, CA).

### Gene expression analysis

Total RNA extracted from PBMCs and adipose tissue using AllPrep RNA/Protein and The RNeasy Lipid Tissue extraction kits, respectively (Qiagen, Inc., Valencia, CA) was converted to cDNA using High Capacity cDNA Reverse Transcription Kit (Applied Biosystems, Foster City, CA) and analyzed by quantitative real-time PCR using Rotor Gene Q-100 (Qiagen, Inc., Valencia, CA). Changes in gene expression of the selected genes between obese and controls subjects were determined by the comparative ΔΔCT method [24] using GAPDH and β-actin as internal references. ΔΔCT = ΔCT (obese group)-ΔCT (control group) for RNA samples.

### Western blot analysis

Western blots were carried out on whole PBMC extracts prepared in RIPA buffer (50mM Tris-HCl pH 7.5, 150mM NaCl, 1% Triton x100, 1mM EDTA, 0.5% Sodium deoxycholate and 0.1% SDS). Cytoplasmic and nuclear extracts were prepared from PBMCs using commercially available kit as recommended by the manufacturer (BioRad, Hercules, CA). Protein concentration was determined by Bradford method using globulin as a standard. For Western blot, 20 µg of proteins were resolved on 10% SDS-PAGE gels. Proteins were then transferred onto PVDF membranes, blocked with 5% non-fat dried milk in Tris-buffered saline containing 0.05% Tween 20 (TBST) for 1 h at room temperature (RT) and then probed with the primary antibody for overnight at 4°C. After washing, the membranes were incubated with horseradish peroxidase-conjugated secondary antibody for 2 h at RT. Finally, protein bands were visualized by chemiluminescence and the images were captured by using the Versadoc 5000 system (BioRad, Hercules, CA). Primary antibodies used in this study consisted of anti-HDAC4 (Catalog # ab1437, Abcam, Cambridge, CA), anti-TSP1 (Catalog # ABIN749558, Antibodies online.com, Atlanta, GA) and anti-HSF1 (Catalog # 4356, Cell Signaling Technology, Inc, Danvers, MA). Tubulin (Catalog # 05-661, Millipore, Timecula, CA) and Lamin B (Catalog # 12586, Cell Signaling Technology, Inc, Danvers, MA) were used as internal controls. For densitometric analysis, the intensity of the bands was determined using Quantity One Software (BioRad, Hercules, CA).

### Immunohistochemistry

Formalin fixed, paraffin embedded adipose tissue samples were prepared and used to make sections for immunohistochemical studies as described previously [25]. Briefly, sections were deparaffinized and the antigens were retrieved at high-temperature using antigen unmasking solution (Dako, Denmark). The endogenous peroxidase was quenched using 3% H_2_O_2_ (Merck Schuchardt, Gemany) for 30 min at RT. Sections were blocked with 5% fat-free milk for 60 min at RT followed by 1% BSA for another 60 min and then, incubated at 4°C for overnight with the corresponding primary antibody. After washing, sections were stained with horseradish conjugated secondary antibody (Dako, Denmark) for 60 minutes at RT. Colors were developed using DAB kit (Dako, Denmark) and sections were counterstained with hematoxylin (Sigma Aldrich, St. Louis, MO). All slides were scanned at 20x magnification and the quantification of the immunohistochemical data was done using Aperio ImageScope software version 11.1 (Aperio, Vista, CA). The algorithm Positive Pixel Count v9 of this software provided the percentage of positive staining (number of stained pixels) as compared to the whole slide. Finally, each annotated picture was manually checked against the original slide picture to ensure the correct matching between the positive staining and the software annotation.

### Plasmid constructs

The expression vector encoding HDAC4 (pCMV-HDAC4) and the pCMV empty vector were purchased from OriGene (OriGene Technologies, Inc, Rockville, MD). Reporter plasmids carrying luciferase gene under the control of NF-κB were described previously [26]. Briefly, plasmids 3xwt-κB-pGL3 and 3xmut-κB-pGL3 were constructed by inserting three copies of wild type (5’-AGTTGAGGGGACTTTCCCAGGCTG-3’) or mutant (5’-AGTTGAATCGACTTTCCCAGGCTG-3’) NF-κB binding site into the unique *Nhe*-I and *Xho*-I restriction sites upstream of the SV40 minimal promoter of pGL3 vector (Promega, Madison, WI).

### Cell culture, transfection and luciferase assays

Human embryonic kidney (HEK-293) cell line was obtained from American Type Culture Collection (Rockville, Baltimore, MD). Cells were cultured in Eagle’s Minimum Essential Medium (EMEM) supplemented with 10% fetal bovine serum and penicillin/streptomycin. For transient transfection assays, cells at ~80% of confluence were transfected with 24 µg of DNA using Lipofectamine method as recommended by the manufacturer (Invitrogen, Carlsbad, CA). Following transfection, cells were incubated in complete EMEM media for 24-36 hours and then, harvested for luciferase assays. Luciferase assays were performed using the Dual Luciferase Assay kit (Promega, Madison, WI). To induce NF-κB, cells were stimulated with 25 ng/ml of TNF-α (R & D Systems, Minneapolis, MN) overnight. Luciferase activity was measured on Synergy Hybrid H4 plate reader (BioTek, Winooski, VT) and normalized according to protein concentration.

### Statistical analysis

Statistical analyses were performed with SAS version 9.2 (SAS Institute, Inc., Cary, NC). Unless otherwise stated, all descriptive statistics for the variables in the study were reported as means ± standard deviation. Non parametric Mann-Whitney t-test was used to determine significance of difference in means between the two groups as indicated in the Figure legends. Correlations between variables were calculated with the Spearman’s rank correlation test. Differences were considered statistically significant at *P*-values less than 0.05.

## Results

### Baseline characteristics of study population

The physical characteristics of the study population baseline are displayed in Table 1. As the population was matched for age, there was no significant age difference between the two groups. As expected, body mass index (BMI), percent body fat (PBF), waist and hip circumferences were all significantly higher in obese subjects than in the control (*P < 0.0001*). Table 2 shows that obese subjects had higher systolic and diastolic blood pressures (*P = 0.03* and *P = 0.05*, respectively). No significant difference between the two groups was observed in the lipid profile as well as the levels of fasting glucose, HbA1C, insulin and C-peptide. By contrast, the levels of leptin and PAI-1 were significantly higher in obese subjects (*P = 0.001* and *P = 0.006*, respectively). Obese subjects had also higher levels of the inflammatory chemokine RANTES (*P = 0.04*), but there was no significant difference with the remaining inflammatory mediators including TNF-α and IL-6 (Table 2 and data not shown). Both lean and obese subjects had comparable levels of ROS, but TBARS levels were significantly higher in obese subjects (*P = 0.006*, Table 2).

### Proteomic Analysis

**Figure 1:**
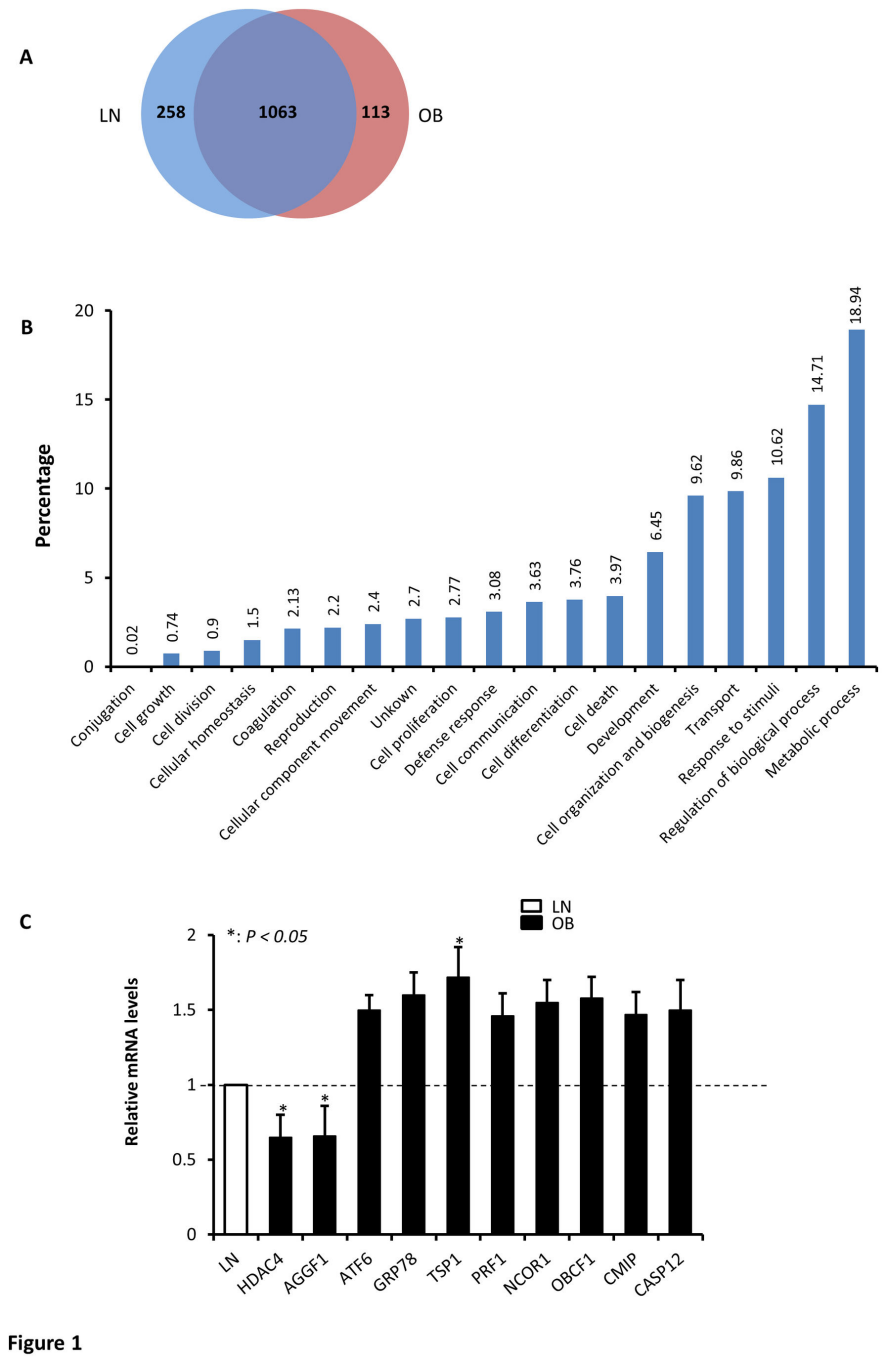
Effect of obesity on protein expression in PBMCs. Venn diagram showing the number of unique and overlapping proteins identified from lean (LN) and obese (OB) subjects (A). Distribution of all the identified proteins according to their biological processes (B). Both panels were generated using ProteinCenter software (Thermo Scientific, Germany). qRT-PCR data carried out on 10 genes to validate the observed differential protein expression at the mRNA levels using total RNA isolated from PBMCs of lean and obese male subjects (n=5 each) and the data are presented as fold changes in obese compared to lean subjects (C). The gene names and primer sequences are shown in Table S6. * *P* < 0.05 as determined using student’s t-test.

In this study, we compared the proteomic profile of PBMCs from lean and obese subjects using a label-free proteomic approach. Samples were subjected to a reverse phase nano-LC coupled to high resolution Orbitrap-Velos MS and protein identification was done by Proteome Discoverer software using Sequest and Mascot search engines against the human international protein index (IPI) database. Relative changes in protein levels between different groups were calculated from the obtained mass spectra using Sieve software. To account for the biological variability, PBMCs samples from lean and obese subjects (n=6 each) were pooled into pairs to make three different biological replicates from each group and each peptide should be identified at least in 2 out of 3 biological replicates as illustrated in the flow chart (Figure S1). All proteins were identified at 1% of false discovery rate. As shown in Figure 1A, a total number of 1434 proteins were identified from the combined MS runs, consisting of 1321 proteins from lean and 1176 proteins from obese, of which, 1063 proteins were found to be common between the two groups (Figure 1A). Figure 1B indicates that the total proteins identified in this study are involved in a wide range of cellular functions including metabolism, regulation of biological process, response to stimuli, transport, and cell organization and biogenesis. The detailed lists of all identified peptides and proteins in both groups are presented in the Tables S1, S2, S3 and S4. Using SIEVE software to analyze differential protein expression levels between the two groups, 47 proteins among the 1063 common proteins shown in Figure 1A were found to be differentially expressed (at least 1.5-fold changes) between lean and obese subjects out of which, 18 proteins were overexpressed in obese and 29 proteins were overexpressed in lean (Table 3). A detailed list of quantified proteins common in both groups is displayed in Table S5.

**Table 3:** List of differentially expressed proteins identified in PBMCs collected from lean and obese subjects.

IPI Number		Protein Description	Symbol	Fold Changes*	SD
IPI00296099.6	Thrombospondin-1		TSP1	↑7.7	0.05
IPI00219757.13	Glutathione S-transferase P		GSTP1	↑2.9	0.17
IPI00029166.4	Homeobox protein		EMX2	↑2.3	0.23
IPI00293423.3	Perforin-1		PRF1	↑2.2	0.12
IPI00645645.1	T-complex 11 homolog		TCP11	↑2.1	0.16
IPI00152658.1	Serine/threonine-protein kinase		NEK7	↑1.9	0.16
IPI00794900.3	cDNA FLJ56016, highly similar to C-1-tetrahydrofolate synthase		MTHFD1	↑1.9	0.24
IPI00010832.1	Cat eye syndrome critical region protein 6		CECR6	↑1.8	0.14
IPI00021739.2	PERQ amino acid-rich with GYF domain-containing protein 1		GIGYF1	↑1.8	0.26
IPI00031016.1	JAK2 Tyrosine-protein kinase		JAK2	↑1.7	0.39
IPI00413436.2	Cytochrome c oxidase assembly protein		COX19	↑1.7	0.19
IPI00003362.2	GRP78 protein		GRP78	↑1.7	0.36
IPI00383810.2	Putative uncharacterized protein DKFZp434O0617		PIAS3	↑1.6	0.36
IPI00007800.1	Angiopoietin-related protein 2		ANGPTL2	↑1.6	0.04
IPI00456750.2	Niban-like protein 1		FAM129B	↑1.6	0.16
IPI00100630.6	Myeloid/lymphoid or mixed-lineage leukemia translocated to 1		MLLT1	↑1.6	0.30
IPI00028438.4	c-Maf-inducing protein isoform		CMIP	↑1.5	0.11
IPI00400922.5	Protein RRP5 homolog		PDCD11	↑1.5	0.24
IPI00022022.1	Polyphosphoinositide phosphatase		Figure 4	↓2.26	0.92
IPI00410380.2	Isoform 2 of Parkin coregulated gene protein		PACRG	↓2.19	0.62
IPI00219806.7	Protein S100-A7		S100A7	↓2.18	0.79
IPI00005685.2	Paraneoplastic antigen Ma1		PNMA1	↓2.14	0.60
IPI00298497.3	Fibrinogen beta chain		FGB	↓2.11	0.57
IPI00010088.2	Histone deacetylase 4		HDAC4	↓2.11	0.66
IPI00414661.7	La-related protein 6		LARP6	↓1.95	0.79
IPI00745814.2	Similar to Cathepsin D precursor LOC100287770;LOC100291562 hypothetical protein XP_002343109			↓1.86	0.62
IPI00427739.1	Isoform 1 of Leucine-rich repeat-containing protein 1		LRRC1	↓1.85	0.73
IPI00915861.1	Putative uncharacterized protein NGEF		NGEF	↓1.71	0.61
IPI00009448.1	Tumor necrosis factor, alpha-induced protein 3		TNFAIP3	↓1.69	0.79
IPI00290462.5	Carbonyl reductase [NADPH] 3		CBR3	↓1.66	0.51
IPI00019502.3	Isoform 1 of Myosin-9		MYH9	↓1.65	0.44
IPI00552787.4	RNA binding motif protein 20		RBM20	↓1.65	0.47
IPI00016868.3	Telomere length regulation protein TEL2 homolog		TELO2	↓1.65	0.49
IPI00295976.6	Isoform 1 of Integrin alpha-IIb		ITGA2B	↓1.65	0.49
IPI00418774.3	Isoform 1 of Coiled-coil domain-containing protein 73		CCDC73	↓1.62	0.48
IPI00400838.3	H_3q26 provirus ancestral Env polyprotein		HERV	↓1.62	0.51
IPI00640721.1	Tyrosine 3-monooxygenase/tryptophan 5-monooxygenase activation protein, beta polypeptide		YWHAB	↓1.61	0.58
IPI00005782.1	Protein phosphatase 1D		PPM1D	↓1.61	0.61
IPI00386324.2	Seven transmembrane helix receptor		GPR142	↓1.60	0.57
IPI00329245.8	Isoform 2 of Ankyrin repeat and LEM domain-containing protein2		ANKLE2	↓1.56	0.43
IPI00869011.2	Caspase recruitment domain-containing protein 11		CARD11	↓1.55	0.70
IPI00742823.1	Similar to DNA-binding protein		LOC283547	↓1.55	0.53
IPI00069208.1	Isoform 1 of Testis-specific chromodomain protein Y-linked, 1B		CDY1B	↓1.53	0.33
IPI00464990.1	Isoform 2 of Platelet glycoprotein 1b beta chain		GP1BB	↓1.52	0.56
IPI00465139.2	Isoform 1 of Stearoyl-CoA desaturase 5		SCD5	↓1.52	0.57
IPI00470491.3	Isoform 2 of Nuclear receptor coactivator 1		NCOA1	↓1.52	0.53
IPI00297550.8	Coagulation factor XIII A chain		F13A1	↓1.51	0.45

* Protein levels are expressed as a fold increase (↑) or decrease (↓) in obese relative to lean group. SD is calculated from 3 independent experiments.

### Validation of the proteomics data by quantitative real-time PCR

In order to validate the above proteomics data, we selected a set of 10 genes among the differentially expressed proteins between lean and obese subjects before exercise and analyzed their expression pattern between lean and obese subjects by quantitative real-time PCR (qRT-PCR). As shown in Figure 1C, there was clear up- and down-regulation in the expression pattern at the mRNA levels between the two groups that was consistent with the proteomics data. Indeed, qRT-PCR data showed a significant reduction in the expression of histone deacetylase 4 (HDAC4) and the angiogenic factor AGGF1 mRNA (*P < 0.05*) and a significant increase in the expression of thrombospondin 1 (TSP1) mRNA (*P < 0.05*). There was also a trend of increase in the expression of the remaining genes in obese subjects; however, this change was not statistically significant (Figure 1C).

### Effect of physical exercise on proteomic profile

**Figure 2:**
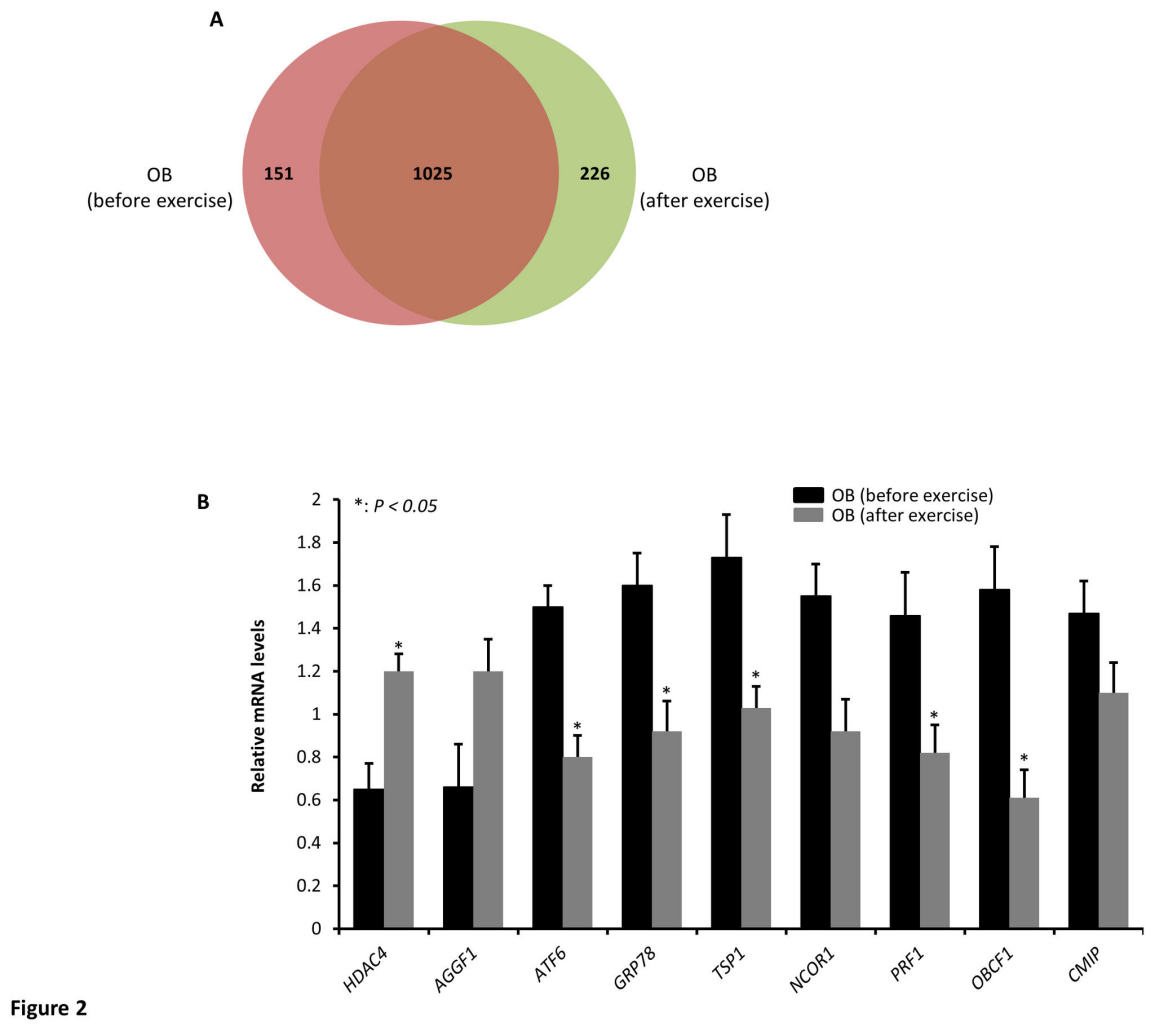
Effect of physical exercise on protein expression in PBMCs of obese subjects. Venn diagram showing the number of unique and overlapping proteins identified from obese before and after 3 months of physical exercise (A). qRT-PCR data carried out on 9 genes to validate the observed differential protein expression at the mRNA levels. Total RNA were isolated from PBMCs of male obese before and after exercise obese (n=5 each) and the data are presented as fold changes in obese compared to lean subjects (B). * *P* < 0.05 as determined using student’s t-test.

Physical exercise is an important component of healthy lifestyle that is highly prescribed as a part of the prophylactic treatment and management of obesity and the clinical manifestations associated with it. These beneficial effects prompted us to investigate if physical exercise has an effect on protein expression levels in obese subjects. For this purpose, differential proteomic expression was carried out on PBMCs collected from obese subjects prior to physical exercise (baseline) and at the end of 3 months of physical exercise program. As shown in Figure 2A, a total of 1402 proteins were identified from the combined MS runs obtained from both groups, of which 1025 proteins were found to be common between the two groups. In addition, 151 proteins were found exclusively in obese before exercise against 226 proteins that were found exclusively in obese subjects that completed the exercise program (Figure 2A). The detailed lists of all identified peptides and proteins in obese after physical exercise are presented in the Tables S7 and S8, respectively. Further analysis indicated that out of the 1025 common proteins between the two groups shown in Figure 2A, 38 proteins were found to be differentially expressed with at least 1.5-fold changes between obese subjects before and after exercise out of which, 17 proteins were increased by exercise and 21 proteins were decreased by exercise (Table 4) and 9 of these differentially expressed proteins were subsequently validated by qRT-PCR (Figure 2B). A detailed list of quantified proteins common in both groups is displayed in Table S9.

**Table 4:** List of differentially expressed proteins identified from PBMCs collected from obese subjects at baseline and after 3 months of physical exercise.

**IPI Number**	**Protein Description**	**Symbol**	Fold Changes*	**SD**
IPI00432360.1	Ubiquitin associated protein 2	UBAP2	↑3.8	0.13
IPI00045478.1	Ras-related protein Rab-40C	RAB40C	↑3.7	0.14
IPI00008711.3	Wolframin	WFS1	↑2.9	0.19
IPI00022597.1	NEDD8-conjugating enzyme Ubc12	UBE2M	↑2.5	0.23
IPI00442274.3	Isoform 1 of NF-X1-type zinc finger protein NFXL1	NFXL1	↑2.4	0.27
IPI00167285.4	Isoform 1 of Cyclin N-terminal domain-containing protein 1	CNTD1	↑2.4	0.17
IPI00010706.1	Glutathione synthetase	GSS	↑1.9	0.24
IPI00855998.1	Centromere protein F	CENPF	↑1.8	0.24
IPI00552375.2	PSMG4 Chromosome 6 open reading frame 86		↑1.7	0.17
IPI00017704.3	Coactosin-like protein	COTL1	↑1.7	0.22
IPI00549955.3	Meiosis-specific nuclear structural protein 1	MNS1	↑1.6	0.34
IPI00169383.3	Phosphoglycerate kinase 1	PGK1	↑1.6	0.26
IPI00514694.1	Procollagen galactosyltransferase 2	GLT25D2	↑1.6	0.16
IPI00010088.2	Histone deacetylase 4	HDAC4	↑1.5	0.28
IPI00305833.3	WD40 repeat-containing protein SMU1	SMU1	↑1.5	0.18
IPI00909631.1	cDNA FLJ52253, highly similar to *Mus musculus* syntaxin 3 transcript variant C, mRNA	STX3	↑1.5	0.35
IPI00375813.3	Putative uncharacterized protein	C10ORF122	↑1.5	0.22
IPI00878979.1	Isoform 2 of Double-strand-break repair protein rad21-like protein 1	RAD21L1	↓2.33	0.67
IPI00793653.1	NCOR1 protein (Fragment)	NCOR1	↓2.27	0.80
IPI00022501.1	Isoform 1 of DnaJ homolog subfamily C member 27	DNAJC27	↓1.97	0.52
IPI00296099.6	Thrombospondin-1	TSP1	↓1.90	0.95
IPI00027971.2	ADP-ribosylation factor-like protein 4D	ARL4D	↓1.85	0.51
IPI00910512.1	cDNA FLJ50382, moderately similar to Integrin alpha-5	ITGA5	↓1.78	0.62
IPI00005565.2	Diacylglycerol kinase theta	DGKQ	↓1.83	0.59
IPI00385917.4	TPCN1 protein	TPCN1	↓1.75	0.61
IPI00175151.7	Isoform 1 of Probable methylcytosine dioxygenase TET2	TET2	↓1.74	0.51
IPI00010415.2	Isoform 1 of Cytosolic acyl coenzyme A thioester hydrolase	ACOT7	↓1.67	0.48
IPI00739386.4	Tyrosine-protein kinase (PRAGMIN)	SGK223	↓1.65	0.56
IPI00869011.2	Caspase recruitment domain-containing protein 11	CARD11	↓1.64	0.48
IPI00893002.1	Isoform 6 of Xin actin-binding repeat-containing protein 2	XIRP2	↓1.61	0.55
IPI00741855.2	Keratin, type I cytoskeletal 39	KRT39	↓1.61	0.61
IPI00856012.1	Isoform 1 of Collagen alpha-6 chain	COL6A6	↓1.61	0.53
IPI00015916.1	Bone-derived growth factor (Fragment)	QSOX1	↓1.59	0.62
IPI00910581.1	cDNA FLJ57960	SARM1	↓1.59	0.47
IPI00298994.6	Talin-1	TLN1	↓1.56	0.19
IPI00010807.1	Frizzled-8	FZD8	↓1.55	0.66
IPI00217393.1	Isoform 1 of Arf-GAP with GTPase, ANK repeat and PH domain-containing protein 2	AGAP2	↓1.5	0.46
IPI00305356.1	Cholesterol 7-alpha-monooxygenase	CYP7A1	↓1.5	0.43

* Protein levels are expressed as a fold increase (↑) or decrease (↓) in obese after exercise relative to obese at baseline. SD is calculated from 3 independent experiments.

**Figure 3:**
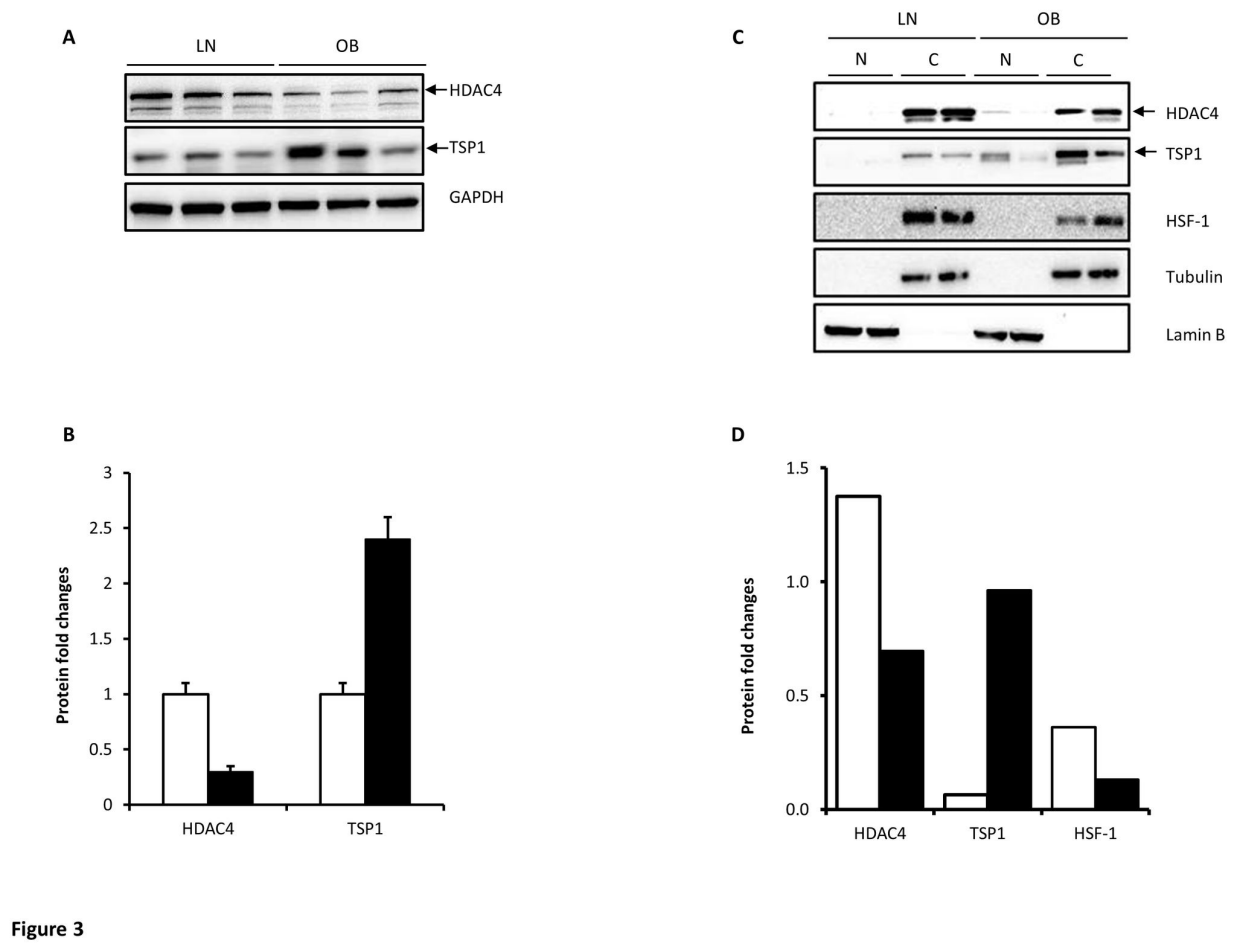
Representative Western blot confirming differential expression of HDAC4 and TSP1 in obese subjects. (**A** and **B**) Total proteins were extracted from PBMC of lean (n=7) and obese (n=7) non-diabetic participants and subjected to western blot using the indicated antibodies. The bands were quantified as described in materials and methods and the relative intensity was determined after correction with GAPDH that was used as internal control to monitor loading efficiency (A). The data are presented in the form of graphs as fold changes compared to lean group (B). (**C** and **D**) Nuclear (N) and cytoplasmic (C) extracts were prepared from PBMCs isolated from lean (LN) and obese (OB) male subjects and subjected to Western blot using the indicated antibodies. The bands representing the cytoplasmic fractions were quantified as described in materials and methods and the relative intensity was determined after correction with Tubulin from the cytoplasmic fraction that was used as internal control to monitor loading efficiency. Lamin B was used as control to monitor nuclear localization (C). The data are presented as fold changes in obese compared to lean subjects (D). The blots shown are representatives of at least two independent experiments with consistent results.

Among the proteins showing dysregulated expression by obesity that was then corrected by physical exercise, we focused on TSP1 and HDAC4 proteins for further investigations. The selection of these protein candidates was also based on their potential therapeutic role as anti-obesity targets. To further confirm the aberrant regulation of HDAC4 and TSP1 in obese subjects, we investigated their endogenous expression by Western blotting using PBMCs extracts from lean and obese subjects. In agreement with proteomic and qRT-PCR data shown above, obese subjects exhibited reduced expression of HDAC4 protein and increased expression of TSP1 protein (Figure 3A and 3B). Given that HDAC4 has been shown to shuttle between nucleus and cytoplasm [27], we particularly analyzed whether obesity triggers a change in its cellular localization. Consistent with the immunoblot data obtained from whole cell lysates, obese subjects showed a reduced expression of HDAC4 protein and increased expression of TSP1 protein than lean subjects (Figure 3C and 3D). Using Lamin B, Tubulin and HSF-1 as internal controls, HDAC4 was predominantly found in the cytoplasm in both groups suggesting that obesity does not trigger a change in the localization of HDAC4.

### TSP1 and HDAC4 are differentially regulated in the adipose tissue of obese subjects

**Figure 4:**
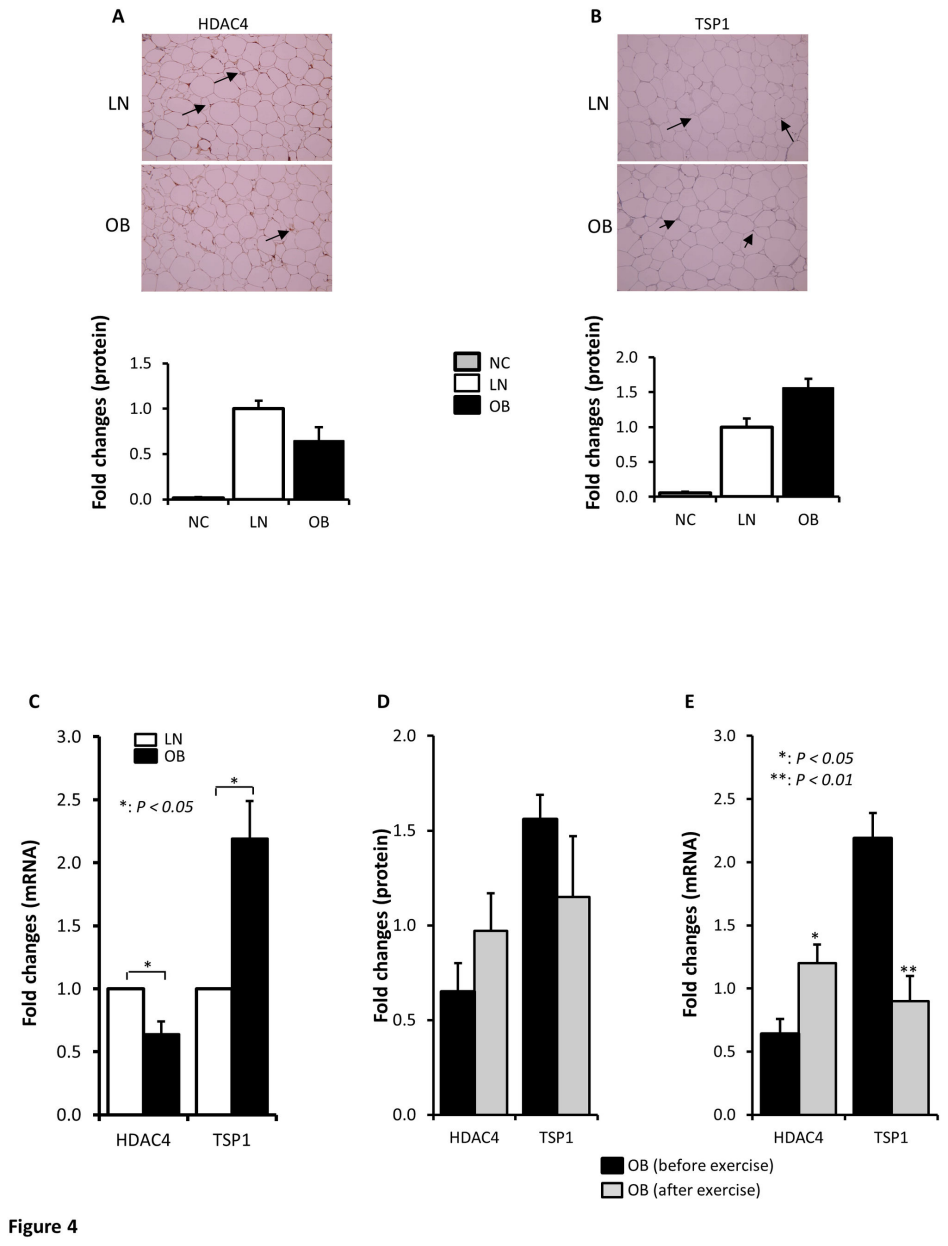
HDAC4 and TSP1 are also differentially expressed in the adipose tissue of obese humans. Representative immunohistochemical (IHC) staining using fat adipose biopsies from lean (n=4) and obese (n=8) male subjects illustrating the expression pattern of HDAC4 (A) and TSP1 (B). Aperio software was used to quantify positive staining (indicated by arrows) and the values are illustrated at the bottom as fold changes compared to lean. As negative control (NC) for the experiment, the primary antibodies were omitted (A, B). qRT-PCR analysis confirming differential expression in of HDAC4 and TSP1 at the mRNA levels in the adipose tissue. Total RNA was extracted from subcutaneous adipose tissue from lean and obese subjects (n=10 each) and analyzed by qRT-PCR. The data are presented as fold changes in obese compared to lean subjects (C). Graphic illustration of IHC quantification of HDAC4 and TSP1 proteins in subcutaneous adipose tissue collected from obese before and after exercise (n=8 each). Aperio software was used to quantify positive staining (D). qRT-PCR analysis showing the effect of physical exercise on the expression of HDAC4 and TSP1 mRNA in the adipose tissue. Total RNA was extracted from subcutaneous adipose tissue from obese subjects before and physical exercise (n=10 each) and analyzed by qRT-PCR. The data are presented as fold changes in obese before exercise compared to obese after exercise (E). * *p*< 0.05 and ** p< 0.01 as determined using student’s t-test.

Adipose tissue is known to play a central role in the development of obesity. We therefore investigated if the changes in the expression of TSP1 and HDAC4 observed in PBMCs occurred also in the adipose tissue. For this purpose, adipose tissue was collected from lean, obese subjects before and after exercise and the relative expression of these proteins was determined by immunohistochemisty (IHC) and qRT-PCR. Consistent with the data observed in PBMCs, IHC data showed trends of reduced expression of HDAC4 (Figure 4A) and increased expression of TSP1 (Figure 4B) in obese compared to lean subjects that were corrected by exercise (Figure 4D). More interestingly, qRT-PCR data shown on Figure 4C indicated a significant decrease in the expression of HDAC4 (*P = 0.046*) and increased expression of TSP1 in obese subjects (*P = 0.011*). Conversely, the expression of HDAC4 was significantly increased in obese subjects after physical exercise intervention (Figure 4E, P *= 0.02*) whereas that of TSP1 was significantly reduced by physical exercise in obese subjects (Figure 4E, P *= 0.001*). Since our study population consisted of males only, we investigated whether there was a gender effect on the expression of HDAC4 and TSP1 by qRT-PCR using adipose tissue. Data obtained from 5 males and 5 females indicated that gender has no effect on differential expression of HDAC4 and TSP1 in obese subjects (Figure S2). Taken together, the initial quantitative proteomics data performed on PBMCs for TSP1 and HDAC4 is in agreement with qRT-PCR and IHC data carried out on adipose tissue.

### Correlation analysis of HDAC4 and TSP1 with clinical profile, inflammatory and metabolic stress markers

To understand the consequence of dysregulated expression of HDAC4 and TSP1 in obese subjects on the physical, clinical and various biochemical parameters, we investigated whether these parameters correlated with the levels of HDAC4 and TSP1 detected in lean and obese subjects before exercise. As shown in Table 5, there were negative correlations that were highly significant between HDAC4 levels and the BMI (r^2^=-0.66; *P < 0.0001*), PBF (r^2^=-0.58; *P = 0.0012*) and RANTES (r^2^=-0.8; *P = 0.001*) and a positive correlation with V_O2 Max_ (r^2^=0.54; *P = 0.024*). There was also a trend of negative correlation between HDAC4 and PAI-1 but it was not significant. No other correlations were found between HDAC4 levels and the remaining parameters measured (Table 5 and data not shown). By contrast to HDAC4, TSP1 positively correlated with the BMI (r^2^=0.54; *P = 0.0014*) and slightly with PBF and PAI-1 but without reaching statistical significance. A trend of negative correlation was also found between TSP1 and V_O2 Max,_ although it was not significant. No correlation was found betweenTSP1 levels and the other parameters (Table 5 and data not shown). To elucidate whether the observed changes in the expression of HDAC4 and TSP1 in obese subjects after physical exercise were concomitant to improvement of physical, clinical and biochemical parameters, we performed a pair wise comparison on 15 obese subjects before and after 3-months exercise to first assess the effectiveness of the exercise program on physical, clinical and biochemical parameters and if so, whether they were consistent with the levels of HDAC4 and TSP1 before and after exercise. Table 6 shows that although there was no significant change in the BMI after 3 months of exercise, there was a significant reduction of PBF, SBP and DBP (*P <0.05*). There was also an improvement of V_O2 Max_ (*P = 0.011*) along with reduced insulin levels (*P = 0.036*) and improved inflammatory response as indicated by reduced levels of the pro-inflammatory IL-6 cytokine and increased levels of the anti-inflammatory IL-10 cytokine (*P = 0.04*). Under these conditions, HDAC4 expression increased significantly (*P = 0.0092*) and TSP1 was reduced significantly by physical exercise (*P = 0.027*) (Table 6).

**Table 5:** Correlation of HDAC4 and TSP1 mRNA expression levels with physical, clinical and biochemical parameters of the study population.

	**HDAC4**					**TSP1**		
	*(r* ^*2*^)	*P-value*					*(r* ^*2*^)	*P-value*
BMI	-(0.66)	<0.0001					0.54	0.0014
PBF	-(0.58)	0.0012					0.34	0.072
V_O2 Max_	0.54	0.024					-(0.42)	0.097
Leptin	-(0.20)	0.29					0.27	0.148
PAI-1	-(0.33)	0.071					0.31	0.09
RANTES	-(0.80)	0.001					0.15	0.923

The correlation was based on ΔΔCT method obtained from qRT-PCR and it was done on 32 subjects consisting of lean (n=13) and obese (n=19) at baseline. Correlation was assessed by using Spearman’s rank correlation coefficient.

**Table 6:** Physical, clinical and biochemical characteristics of obese population (n=15) before and after exercise.

	**Before exercise**	**After exercise**	***P-value***
Age (year)	49.47 ± 13.61	-	-
BMI (Kg/m^2^)	33.74 ± 3.04	33.37 ± 2.47	0.36
PBF (%)	33.89 ± 2.98	33.11 ± 3.94	0.047
Resting HR (beats/min)	73.78 ± 7.60	77.33 ± 11.87	0.39
SBP (mmHg)	131.67 ± 12.25	122.22 ± 8.83	0.04
DBP (mmHg)	86.00 ± 9.66	78.89 ± 3.33	0.043
V_O2 Max_ (ml/kg/min)	18.8 ± 5.35	21.37 ± 5.09	0.011
Cholesterol (mmol/l)	4.88 ± 0.99	4.84 ± 1.01	0.98
HDL (mmol/l)	1.09 ± 0.24	1.03 ± 0.27	0.73
LDL (mmol/l)	3.05 ± 0.84	3.14 ± 0.97	0.63
TG (mmol/l)	1.60 ± 0.83	1.49 ± 0.56	0.58
Glucose (mmol/l)	5.73 ± 1.12	5.78 ± 0.48	0.85
HBA1C (%)	5.96 ± 0.56	5.92 ± 0.46	0.57
C-peptide (ng/ml)	3.17 ± 1.46	2.48 ± 0.73	0.30
GLP-1 (ng/ml)	2.22 ± 1.24	1.95 ± 0.40	0.59
Insulin (ng/ml)	2.88 ± 2.29	1.98 ± 1.79	0.036
Leptin (ng/ml)	7.03 ± 3.04	6.34 ± 3.47	0.9
PAI-1 (ng/ml)	4.01 ± 0.83	3.21 ± 1.38	0.31
TNF-α (pg/ml)	23.28 ± 8.93	19.20 ± 9.19	0.55
IL-6 (pg/ml)	4.38 ± 1.10	3.26 ± 1.02	0.04
IL-10 (pg/ml)	1.87 ± 1.70	2.64 ± 1.67	0.04
IP-10 (ng/ml)	0.48 ± 0.15	0.50 ± 0.21	0.19
RANTES (ng/ml)	1.60 ± 0.65	1.81 ± 0.59	0.21
TBARS (μM)	1.61 ± 0.56	1.45 ± 0.31	0.21
HDAC4 (ΔΔCT)	0.66 ± 0.28	1.19 ± 0.47	0.0092
TSP1 (ΔΔCT)	1.48 ± 0.52	0.74 ± 0.29	0.027

Data are presented as mean ± SD. Paired t-test was used to compare differences in obese before and after 3 months of physical exercise.

### Network analysis

The chronic conditions associated with obesity such as low grade inflammation, hyperlipidemia and dysregulation of the stress response promoted us to investigate whether proteins identified from this work are connected to each other within interaction networks of cellular systems. Using Ingenuity Pathways Analysis software, HDAC4 and TSP1 (known also as THBS1) were found to be indirectly interconnected and involved in important components of functional network (Figure S3). Of particular interest, we found a direct crosstalk between HDAC4 and NF-κB; the master regulator of the immune response pathway.

### Ectopic expression of HDAC4 abolished TNF-α-mediated NF-κB activation in functional assays

**Figure 5:**
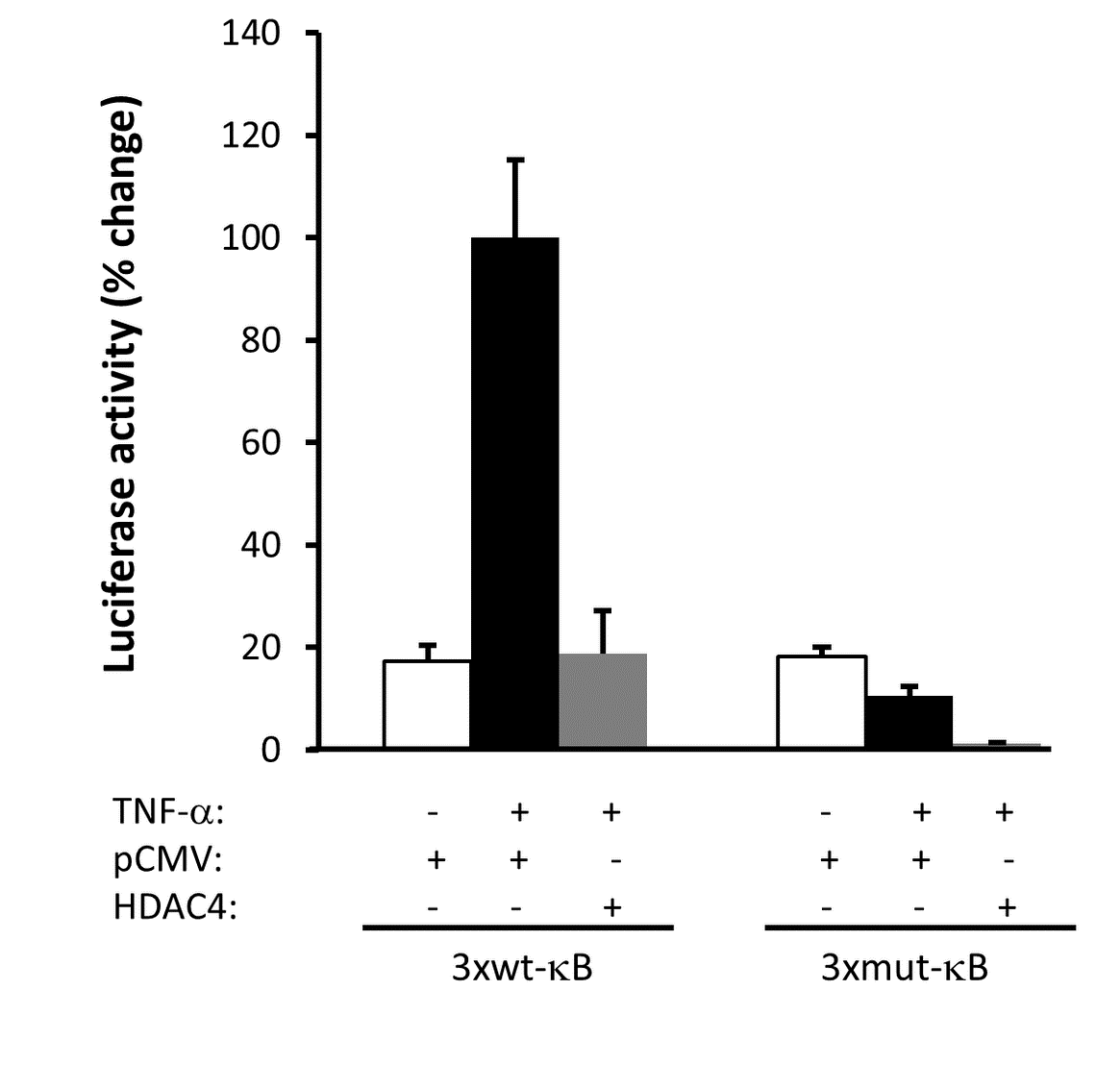
Ectopic expression of HDAC4 impaired NF-κB activation by TNF-α in luciferase assays. Reporter and expression vectors were used in transient transfection assays in HEK293 cells as indicated in materials and methods. Reporter vector in which the firefly luciferase gene is under the control of 3 copies of wild type (3xwt-κB) or mutant (3xmut-κB) NF-κB binding was cotransfected with HDAC4 expression vector. pCMV was used a control empty vector. 24 hours post-transfection, cells were treated with 25 ng/ml of TNF-α for overnight and then, the luciferase activity was carried out as described in materials and methods. Protein concentration was used for normalization.

In order to complement the *in vivo* data shown above, we undertook a series of *in vitro* experiments using cell lines. The decrease of HDAC4 in obese and its restoration by physical exercise is suggestive of a protective role of HDAC4 against obesity. Based on the negative correlation between HDAC4 levels and various metabolic and inflammatory markers (Table 5) and given the importance of stress kinases such as JNK, IKKβ in obesity and insulin resistance, we sought to determine whether overexpression of HDAC4 has an effect on JNK and NF-κB activation. For this purpose, HEK-293 cells were transfected with pCMV-HDAC4 and investigated in our initial attempt the status of JNK phopshorylation following its activation by palmitate. Under our experimental conditions, we failed to obtain consistent results supporting the link between JNK and HDAC4. However, our data demonstrated that overexpression of HDAC4 impaired NF-κB activation by TNF-α in luciferase assays (Figure 5). Our data are in line with the network analysis shown in Figure S3.

## Discussion

The biological underpinnings of obesity are multiple and complex. Although, the contributing roles of several behavioral, environmental and genetic factors to obesity are well established, the molecular mechanisms are still not fully elucidated as obesity and its co-morbidities associated are continuing to rise at an escalating rate. Previous proteomics profiling studies using two major intra-abdominal fat depots, namely the subcutaneous and omental fat tissues have led to the identification of a limited number of differentially expressed proteins in obese subjects in comparison to lean [22]. Although these studies were pivotal in shedding initial light into the mechanisms involved in obesity and its complications, the limited number of identified proteins was, in large part due to the inherent limitation of the technology (2D gel combined to Maldi-Tof) and the technical challenges to extract soluble proteins from fat adipose tissue. To complement these studies, we used peripheral blood mononuclear cells (PBMCs) as a surrogate tissue in our label-free LC-MS shotgun screening effort as it provides a large pool of expressed protein with the potential to be differentially regulated in obese subjects. We report here the identification and quantification of 47 proteins that were differentially regulated between lean and obese subjects. Furthermore, we investigated the expression profiling pattern in obese subjects before and after a defined physical exercise program and we identified and quantified 38 proteins that showed differential expression before and after physical exercise in obese subjects. To the best of our knowledge, this study provides the first large scale quantitative proteomics snapshot on human obesity from PBMCs between lean and obese subjects. It also describes the effect of physical exercise on differential protein expression in obese subjects. Among the differentially expressed protein between lean and obese subjects, we focused on TSP1 and HDAC4 as they may represent potential targets against obesity. The selection of these proteins was based on the fact that they were among the proteins dysregulated in obese subjects and then, corrected by physical exercise.

TSP1 is a multifunctional adipokine that has been shown to be involved in the regulation of various pathways such as angiogenesis, cell proliferation as well as inflammation and wound healing [28,29]. In our current investigation, we found that TSP1 protein and mRNA are upregulated in obese subjects in both PBMCs and adipose tissue. Our findings on TSP1 are consistent with previous studies that associated TSP1 with obesity and insulin resistance [29,30,31]. In agreement with previous studies [29,32], the observed increase of TSP1 in our study population correlated also positively with BMI and slightly with PBF. Furthermore, our data showed for the first time that obesity-induced dysegulation of TSP1 mRNA and protein was corrected by regular physical exercise in both PBMCs and adipose tissue. The significant attenuation observed in TSP1 levels following physical exercise and the improvement of clinical outcomes associated with obesity for the participating group confirmed the hypotheses that physical exercise can provide an effective approach for combating the deleterious effects associated with obesity. The direct effect of TSP1 on the inflammatory and metabolic profiles was recently reported using diet-induced obesity in TSP1 KO mice [30]. Indeed, this study clearly demonstrated that, although TSP1 deficiency does not prevent the development of high fat diet induced obesity, those animals displayed reduced inflammatory response and improved glucose and insulin homeostasis as compared to obese wild type mice [30]. In their model, the improved glucose tolerance and insulin sensitivity were associated with reduced accumulation of pro-inflammatory macrophages in adipose tissue and decreased levels of IL-6 and TNF-α [30]. In addition, it has been suggested that TSP1 may contribute to obesity-induced metabolic inflammation by modulating other immune cells such as T cells through TGF-β signaling pathway [30]. In our study, the increase of TSP1 in obese subject was concomitant with increased circulating levels of PAI-1; a downstream target of TGF-β that was shown in previous studies to be associated with obesity and inflammation [33,34]. This suggests that TSP1 is contributing to systemic and local inflammation driven by obesity. Taken together, the increased expression of TSP1 in obese subjects, its restoration by physical exercise and its significant correlation with metabolic, inflammatory and stress markers suggest that TSP1 may represent a potential target to modulate the chronic inflammatory and metabolic abnormalities associated with obesity.

HDAC4, a protein involved in histone acetylation and chromatin remodeling was another potential target that we identified and validated in the current study. Our data demonstrated for the first time a reduced expression of HDAC4 mRNA and protein in human obese subjects both in PBMCs and adipose tissue. This is consistent with clinical data in humans that associated the haploinsufficiency of HDAC4 with obesity [35,36]. In our study, the decrease of HDAC4 mRNA correlated positively with V_O2 Max_ but negatively with BMI, PBF and circulating levels of the pro-inflammatory chemokine RANTES. Future studies using HDAC4 KO animals and/or pharmacological inhibitors are needed to confirm these initial observations. Another important finding of our study is the restoration of the normal expression of HDAC4 in obese subjects after a physical exercise program even though no major change in the BMI was observed. Taken together, the significant decrease of HDAC4 in obese subjects, its negative correlation with anthropometric and metabolic markers and the restoration of its normal expression after a defined exercise program suggest that HDAC4 may play a protective role in obesity and most likely in insulin resistance and type 2 diabetes. These findings provided further evidence that approaches leading to enhanced expression of HDAC4 or its activity may be used to mitigate metabolic stress triggered by obesity. There is a widespread interest in studying protein acetylation because of their critical role in regulating gene expression and their active involvement in a number of metabolically related disorders such as obesity, insulin resistance, diabetes and cardiovascular diseases [37,38,39,40]. The recent investigation that documented the existence of 3600 lysine acetylation sites on 1750 proteins highlights the importance of this post translational modification [41]. HDAC proteins regulate histone acetylation in a sequence-specific manner to repress and in some circumstances to activate various transcription programs [42,43,44]. HDACs are also known for their ability to modulate various biological processes by targeting other non-histone proteins such as NF-κB [45,46], HSF-1 [47], STAT3 [48] and the insulin receptor substrate-1 (IRS-1) [49]. Pioneer studies on the role of HDACs proteins in modulating various cellular responses were made available by using HDAC KO animals or HDAC inhibitors. For instance, HDAC4 was shown to play an anti-stress role in response to calorie restriction [50,51] as well in promoting cell survival [52] and interfering with apoptosis [53]. In the context of obesity, HDAC4 was also recently reported to be involved in regulation of GLUT4 during adipocyte differentiation [54] as well as promoting lypolysis [55]. Likewise, preadipocytes from HDAC9 KO mice exhibited accelerated adipogenic differentiation and thus, demonstrating its direct role as a negative regulator of adipogenesis [56]. The ubiquitin-binding HDAC6 was reported to play a crucial role in protein homeostasis “proteostasis” following accumulation of misfolded and aggregated proteins [57]. This role is executed, at least in part, through interaction with components of the heat shock response [58]. HDAC3 was also shown to display a protective role against shear stress-induced endothelial cells and vasculature injury [59]. Impaired expression of HDAC2 was associated with induction of cell senescence in a manner that depends on p53 [60].

HDAC proteins are known to shuttle between nucleus and cytoplasm by a mechanism that involves AMPK, PDK and CamKII [52,61,62]. In our case, HDAC4 was found mainly in the cytoplasm of PBMCs isolated from both lean and obese subjects, suggesting that obesity does not trigger any change in cellular localization of HDAC4. Our data are in agreement with previous studies that reported a cytoplasmic localization of HDAC4 in endocrine cells [61] and retinal cells [52]. In the skeletal muscle however, HDAC4 was predominately found in the nucleus but exported immediately after exercise in a manner that was concomitant with the activation of AMPK and CaMKII kinases [27]. A recent study reported that DNAJB5 cochaperone acts as a nucleocytoplasmic shuttling of HDAC4 during oxidative stress [58]. This role is consistent with the findings that HDAC4 interacts with various members of DNAJ family including DNAJB5 and such interaction is important for maintaining proteostasis and protection against stressful conditions [47,58,63,64]. The cytoplasmic role of HDAC4 in PBMCs, if any, remains to be elucidated, nevertheless, the pro-survival role of HADC4 on retinal cells was mediated in part by activation of hypoxia inducible factor 1α [52]. Our results indicated that HDAC4 suppresses TNF-α-mediated NF-κB activation (Figure 5) and such effect is supported by the predicted network analysis (Figure S3). Previous studies reported that obesity and insulin resistance are associated with impaired expression of various components of the heat shock response, including members of DNAJ family [6,65,66]. It is also known that physical exercise induces the heat shock response and improves the clinical parameters associated with obesity and insulin resistance [13,67]. In our current investigation, we also showed that exercise induced the expression of HDAC4. On the other hand, HADC4 regulates proteostasis via interaction with and deacetylation of members of DNAJ family [58] through a mutual crosstalk between HDAC4 and component of the heat shock response [58]. Future investigations focusing on the role HSF-1, a master regulator of the heat shock response on the expression of HDAC4 should elucidate such relationships. Our finding on HDAC4 raised a series of questions that needs to be investigated in future follow-up studies. For instance, what is the status of histone acetylation in obese subjects? What are the molecular mechanisms governing the observed downregulation of HADC4 in obese subjects and how does exercise restores the normal expression of HDAC4? In addition, are HDAC4 KO mice more prone to obesity and insulin resistance?

There are limitations in this study that deserve consideration. The number of subjects used to carry out the proteomic profiling was small. In addition, despite the fact that both male and female are prone to obesity, the study analyzed differential protein expression in adult male subjects only and this may not reflect the global changes in females due to hormonal regulation and menopause as well as in younger population. However, despite these limitations, the present study showed clearly that obesity triggers differential regulation of TSP1 and HDAC4 proteins at protein and mRNA levels in both PBMCs and adipose tissue.

In summary, our proteomic approach performed on obese humans before and after physical exercise revealed HDAC4 as target that may have a potential therapeutic benefit for the control and management of obesity and insulin resistance. Our study documented also the beneficial effect of exercise in modulating the expression of HDAC4 and TSP1.

## Supporting Information

Figure S1 **Workflow of the proteomic analysis using PBMC samples from human subjects.**
(TIF)

Figure S2 **Validation of the proteomics data by qRT-PCR.** qRT-PCR analysis showing the difference in expression level of HDAC4 and TSP1 between male and female obese subjects at the mRNA levels in adipose tissue. Total RNA was extracted from subcutaneous adipose tissue from 5 males (M) and 5 females (F) indicated that gender has no effect on differential expression of HDAC4 and TSP1 in obese subjects. The data are presented as fold changes in obese compared to lean subjects.
(TIF)

Figure S3 **Network analysis.** Protein network analysis showing the link between HDAC4 and other proteins including TSP1 (known also as THBS1*) and NCOR1.
(TIF)

Table S1 **List of peptides identified in lean subjects.**
(XLSX)

Table S2 **List of proteins identified in lean subjects.**
(XLSX)

Table S3 **List of peptides identified in obese subjects at baseline.**
(XLSX)

Table S4 **List of proteins identified in obese subjects at baseline.**
(XLSX)

Table S5 **List of proteins quantified by Sieve 1.3 software and shown as ratios of protein expression between lean and obese subjects at baseline.**
(XLSX)

Table S6 **List of primers used for qRT-PCR.**
(DOCX)

Table S7 **List of peptides identified in obese subjects after 3 months of physical exercise.**
(XLSX)

Table S8 **List of proteins identified in obese subjects after 3 months of physical exercise.**
(XLSX)

Table S9 **List of proteins quantified by Sieve 1.3 software and shown as ratios of protein expression between obese before and after exercise.**
(XLSX)
