# Supplementary figures and images for: Proteomics Analysis of Human Obesity Reveals the Epigenetic Factor HDAC4 as a Potential Target for Obesity
Source: PLoS One. 2013 Sep 24;8(9):e75342. doi: 10.1371/journal.pone.0075342 (PMC3782461; doi:10.1371/journal.pone.0075342)

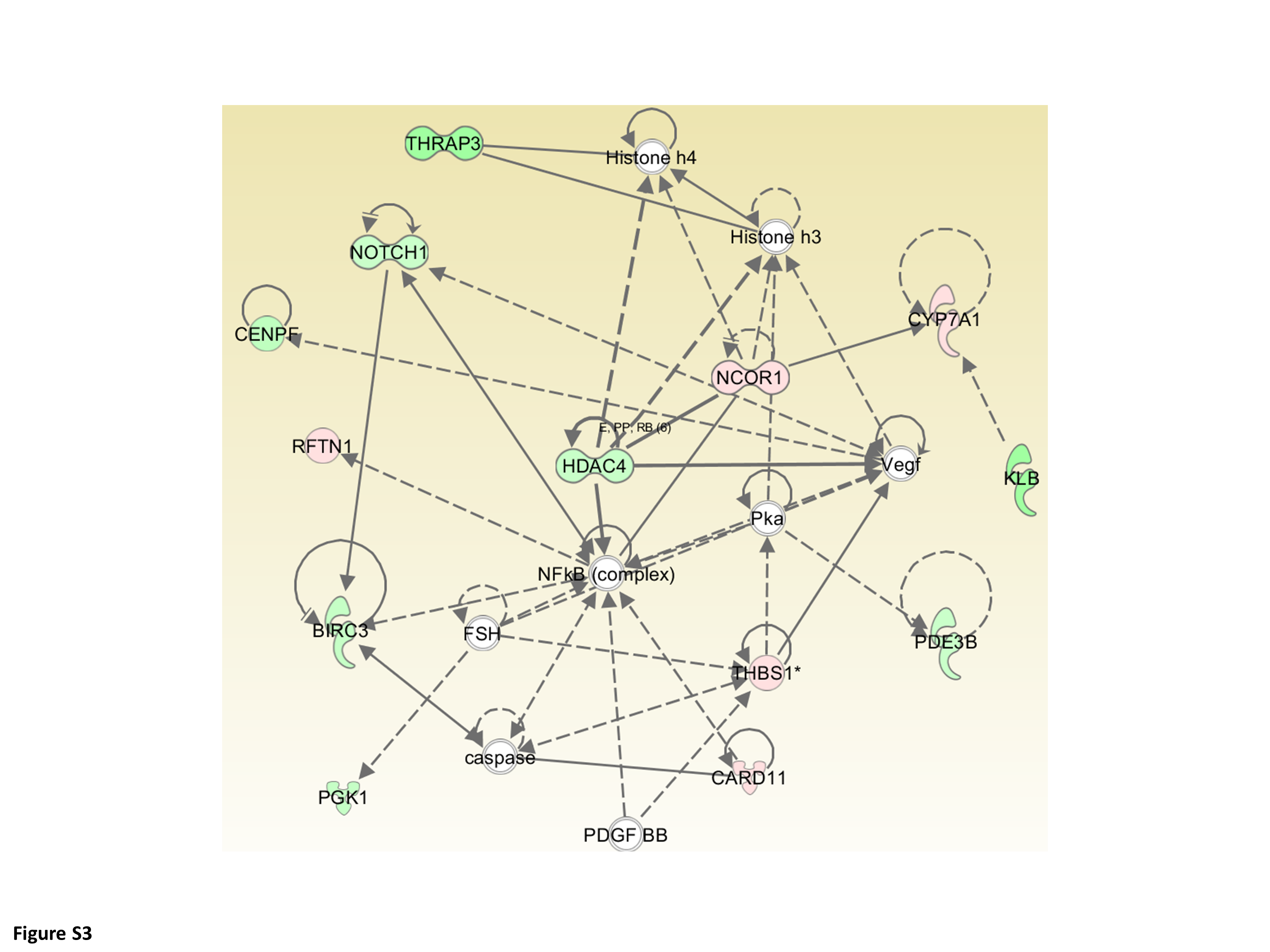

Supplement: Figure S3 — Network analysis. Protein network analysis showing the link between HDAC4 and other proteins including TSP1 (known also as THBS1*) and NCOR1. (TIF) [file pone.0075342.s003.tif]
