# Supplementary material for: Proteomics Analysis of Human Obesity Reveals the Epigenetic Factor HDAC4 as a Potential Target for Obesity
Source: PLoS One. 2013 Sep 24;8(9):e75342. doi: 10.1371/journal.pone.0075342 (PMC3782461; doi:10.1371/journal.pone.0075342)
Supplement: Table S6 — List of primers used for qRT-PCR. (DOCX) [file pone.0075342.s009.docx]

**Table S6: Primer sequences used for quantitative real time PCR to validate the proteomics data at the mRNA level.**

| Gene name | Symbol | Forward Primer (5’ to 3’) | Reverse Primer (5’ to 3’) |
| --- | --- | --- | --- |
| *Thrombospondin 1* | *TSP1* | -CATGCCACGGCCAACAA- | -TGGCCCAGGTAGTTGCACTT- |
| *Histone deacetylase 4* | *HDAC4* | -CAGGAGATGCTGGCCATGAA- | -GCACTCTCTTTGCCCTTCTC- |
| *Oligonucleotide/oligosaccharide-binding fold-containing protein 1* | *OBFC1* | -GCTACAAGTGTCCCATCTAGATG- | -AAGAGGGAAGGGGTCTCCTC- |
| *Perforin-1* | *PRF1* | -CACCCTCTGTGAAAATGCCCTAC- | -TCCAGTCGTTGCGGATGCTAC- |
| *c-Maf-inducing protein* | *CMIP* | -TGTCTTACTGCAGGTGGTGC- | -GTGTCTGCTGCTCCCTCATT- |
| *Caspase 12* | *CASP12* | -ATCTCACAGCTCAGGAAATGG- | -GCAGTTACGGTTGTTGAA- |
| *Angiogenic factor 1* | *AGGF1* | -AAGGCCAAAATAGGCATTCAT- | -CACTGGTAATGGCTTCGTCA- |
| *Activating transcription factor 6* | *ATF6* | -ACCCACTAAAGGCCAGACG- | -CCACGTGATTAGGGAGCTGT- |
| *Glucose regulated protein 78* | *GRP78* | -CCACCTCAGTCTCCCAGCTAA- | -GCCGAGCATGGTGGTAACA- |
| *Nuclear receptor co-regulator* | *NCOR1* | -CAGAAACCAGCAGCAAATTGC- | -GGAGTCTTCTTTTTCATCTTTTTCC- |
| *Glyceraldehyde phosphate dehydrogenase* | *GAPDH* | -AGGGCTGCTTTTAACTCTGGT- | -CCCCACTTGATTTTGGAGGGA - |
